# Is It Too Late? Machine Learning for Predicting Orchiectomy Versus Testicular Preservation in Acute Torsion

**DOI:** 10.3390/diagnostics16132034

**Published:** 2026-06-29

**Authors:** Onursal Varlikli, Ozan Can Tatar, Mustafa Alper Akay, Semih Metin, Fahriye Nur Cuce, Gulsen Ekingen Yildiz

**Affiliations:** 1 Department of Pediatric Surgery, Faculty of Medicine, Kocaeli University, Umuttepe Yerleskesi, İzmit 41001, Kocaeli, Turkey; pedcerr@gmail.com (M.A.A.); semihmetinn@gmail.com (S.M.); fahriyenurcuce64@gmail.com (F.N.C.); gyekingen@gmail.com (G.E.Y.); 2 Department of Information Systems Engineering, Faculty of Technology, Kocaeli University, Umuttepe Yerleskesi, İzmit 41001, Kocaeli, Turkey; ozancantatar@gmail.com; 3 Department of General Surgery, Kocaeli City Hospital, İzmit 41050, Kocaeli, Turkey

**Keywords:** spermatic cord torsion, machine learning, orchiectomy, reperfusion injury, hematologic parameters

## Abstract

**Objectives:** Testicular torsion is a time-critical pediatric urological emergency in which delayed presentation may increase the likelihood of orchiectomy. However, preoperative estimation of the immediate intraoperative outcome remains challenging. This study aimed to develop and internally validate machine-learning models integrating ischemic timing variables and complete blood count-derived inflammatory indices to predict orchiectomy versus testicular preservation at index surgical exploration in pediatric and adolescent testicular torsion. **Methods:** We retrospectively analyzed 165 children and adolescents who underwent surgical exploration for confirmed testicular torsion. The primary endpoint was orchiectomy at index exploration versus testicular preservation through detorsion and/or orchiopexy. Clinical timing variables and complete blood count-derived indices, including neutrophil-to-lymphocyte ratio, platelet-to-lymphocyte ratio, lymphocyte-to-monocyte ratio, white blood cell-to-monocyte ratio, monocyte-to-mean platelet volume ratio, hemoglobin-to-monocyte ratio, systemic inflammatory response index, and aggregate index of systemic inflammation, were evaluated. Five supervised machine-learning algorithms—logistic regression, random forest, XGBoost, LightGBM, and support vector machine—were assessed using nested stratified cross-validation with an outer 5-fold loop and inner 3-fold hyperparameter selection. Model performance was estimated from out-of-fold predictions. **Results:** Orchiectomy was performed in 37 patients (22.4%), whereas testicular preservation through detorsion and/or orchiopexy was performed in 128 patients (77.6%). Symptom duration was significantly longer in the orchiectomy group than in the preservation group (48.00 [30.00–72.00] vs. 6.00 [2.00–24.00] h, *p* < 0.001). Monocyte count was also higher in the orchiectomy group (0.92 [0.68–1.23] vs. 0.65 [0.50–0.93] × 10^9^/L, *p* < 0.001). Among the combined models, XGBoost achieved the highest numerical discrimination, with a ROC-AUC of 0.926 ± 0.066 and a bootstrap 95% confidence interval of 0.856–0.966. Feature-block and interpretability analyses identified symptom duration as the dominant predictor, followed by emergency department waiting time and selected monocyte-centered inflammatory indices. **Conclusions:** Immediate intraoperative orchiectomy in pediatric and adolescent testicular torsion was most strongly associated with prolonged symptom duration, while selected complete blood count-derived inflammatory indices provided complementary risk information. The combined XGBoost model showed strong internal discrimination and clinically interpretable predictor patterns. However, the model was internally validated only and requires external validation before clinical implementation.

## 1. Introduction

Testicular torsion (TT) is a common pediatric urological emergency, accounting for approximately 10–15% of acute scrotal presentations in male children [1]. This condition occurs when the spermatic cord undergoes torsion, leading to ischemia and posing a risk of testicular loss. Prompt detorsion is crucial; intervention within first 6 h results in high salvage rates (>90%), whereas viability significantly diminishes with delays [1,2,3]. A recent systematic review indicated that testicular survival was approximately 90.4% if surgical intervention occurred within 12 h but decreased to ~54% for delays of 13–24 h and ~18% beyond 24 h [4]. In clinical practice, late presentations often necessitate an orchiectomy. Notably, Lee et al. documented an orchiectomy rate of 41.9%, whereas a systematic review by McDonald et al. indicated an average orchiectomy rate of 49% in surgically treated cases of testicular torsion [5,6]. In practice, minimizing the “door-to-detorsion” time is essential, as each hour of ischemia substantially worsens the prognosis.

Given that clinical assessment and Doppler ultrasound are not infallible, researchers have explored preoperative biomarkers to enhance prognostication. Jang et al. observed that 93.3% of testes were salvaged when symptoms persisted for <3 h, compared with only 26.6% salvage in the 3–12 h window [7]. These findings have generated interest in utilizing routine laboratory tests to evaluate systemic inflammatory indicators. Complete blood count (CBC) tests are cost-effective and widely accessible diagnostic tools. If CBC parameters are correlated with testicular viability, they could serve as practical and easily implementable instruments in the clinical decision-making process regarding the potential outcomes of scrotal exploration in patients with testicular torsion (TT). Notably, derived CBC ratios have demonstrated potential as objective predictors of ischemic injury. The NLR has been independently correlated with torsion outcomes; indeed, a large multicenter pediatric study identified NLR as the most robust predictor of orchiectomy or eventual testicular atrophy following torsion [7]. Other inflammation-based indices, such as PLR and lymphocyte-to-monocyte ratio, have also been suggested as potential markers of irreversible torsion [8,9]. In a study conducted by He et al., mean platelet volume (MPV) was identified as an independent risk factor for the need for orchiectomy. This finding indicates that MPV levels may be correlated with the severity of testicular ischemia and tissue damage [10]. Kikkawa et al. demonstrated that an elevated neutrophil-to-lymphocyte ratio (NLR) serves as a robust predictive marker for diagnosing testicular torsion. Furthermore, they found that integrating this marker with the TWIST score significantly enhanced the diagnostic accuracy [11]. In a study conducted by Yu et al., it was demonstrated that elevated preoperative neutrophil-to-lymphocyte ratio (NLR) values are associated with an increased risk of orchiectomy and reduced testicular viability in cases of testicular torsion [12]. These CBC-derived parameters may fluctuate with the duration of ischemia and the body’s immune response, providing quantitative insights into tissue viability.

Building upon these insights, machine learning (ML) presents a method for integrating diverse clinical factors and laboratory data to enhance prognostic accuracy. Recent studies employing ML models in pediatric torsion have demonstrated high accuracy; for instance, a proof-of-concept study utilized decision trees and logistic regression on postoperative follow-up data, achieving an overall accuracy of approximately 90.5% and confirming a strong association between shorter symptom duration and testicular viability [13]. Such models can generate an objective “risk score” by evaluating time-to-intervention alongside hematologic markers. In our study, we similarly employed ML techniques to integrate ischemic timing variables with CBC indices and other clinical data to predict whether a torsed testis will remain viable or necessitate orchiectomy. Ischemic duration variables, complete blood count (CBC) indices, and clinical parameters were assessed using a comprehensive methodology employing machine learning (ML) algorithms to predict the probability of maintaining testicular viability and the necessity for orchiectomy. The predictive models developed in this study are intended to augment current clinical evaluations in the management of acute scrotal conditions, offering clinicians a robust decision-support tool for critical decision-making by facilitating early risk stratification in cases of testicular torsion, where timely intervention is crucial. Given the irreversible consequences associated with testicular loss, the early identification of high-risk patients is of substantial clinical importance for optimizing surgical planning and ensuring that patients and their families are accurately informed about potential surgical outcomes. Moreover, this approach contributes to the development of a practical, cost-effective, and objective predictive model for routine clinical practice by integrating readily accessible laboratory parameters and advanced analytical techniques. In this context, our study provides an innovative clinical perspective aimed at enhancing the diagnostic accuracy of testicular torsion, reinforcing an organ-preserving strategy, and preventing unnecessary delays.

## 2. Materials and Methods

### 2.1. Study Design and Population

This retrospective observational cohort study aimed to develop and internally validate machine-learning models for predicting the immediate intraoperative surgical outcome in pediatric and adolescent patients undergoing surgical exploration for suspected testicular torsion. The primary endpoint was defined as orchiectomy at index surgical exploration versus testicular preservation through detorsion and/or orchiopexy at index exploration. Patients who underwent orchiectomy were coded as the event group, whereas patients managed with detorsion, orchiopexy, or testicular preservation were coded as the reference group.

The study was conducted at a tertiary care university hospital functioning as a regional referral center between January 2015 and October 2025. The study protocol was approved by the Kocaeli University Clinical Research Ethics Committee (Date: 20 November 2025 Decision No.: KÜ GOKAEK-2025/23/34 and Project No.: 2025/607). All procedures adhered to the ethical principles outlined in the Declaration of Helsinki. Before analysis, patient identifiers were removed and the dataset was anonymized. The study was reported in accordance with the Transparent Reporting of a Multivariable Prediction Model for Individual Prognosis or Diagnosis (TRIPOD) recommendations.

**Figure 1:**
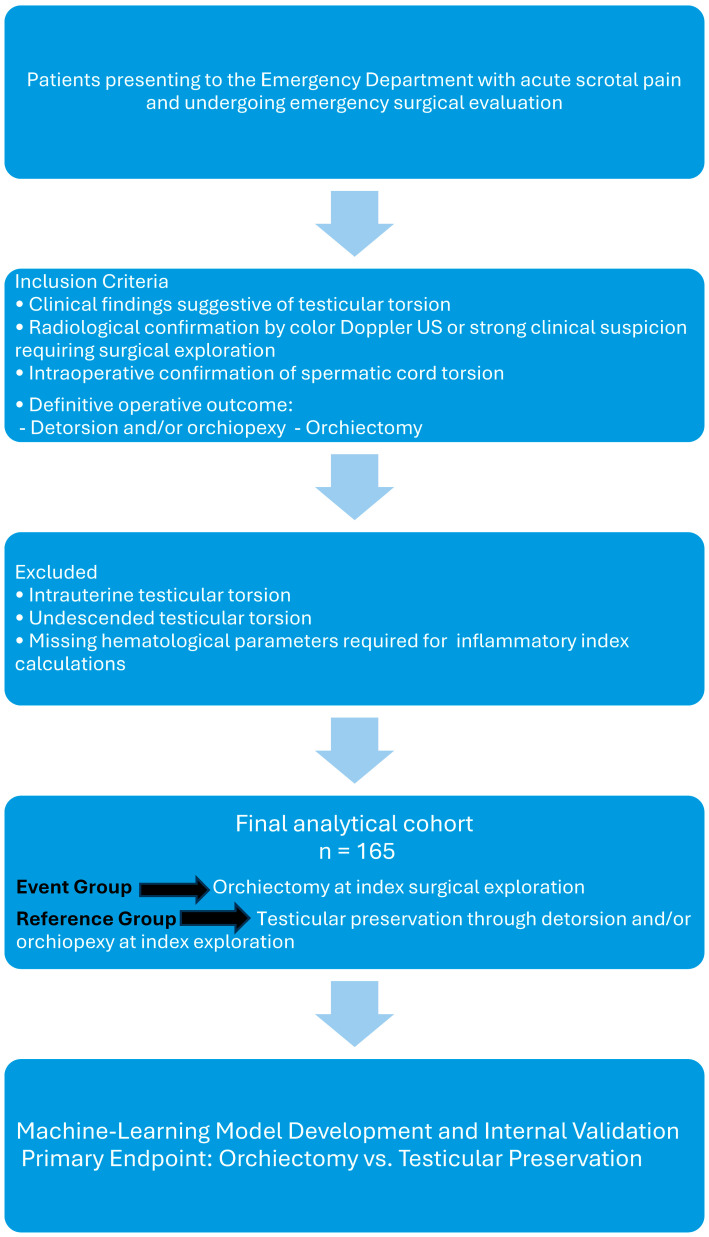
Study flowchart showing patient selection, eligibility assessment, exclusion criteria, cohort formation, outcome classification, and machine-learning model development for prediction of orchiectomy versus testicular preservation in acute testicular torsion.

Patients who presented to the emergency department with acute scrotal pain and subsequently underwent emergency surgical evaluation were assessed for eligibility. The inclusion criteria were clinical findings suggestive of testicular torsion, radiological confirmation by color Doppler ultrasonography or strong clinical suspicion requiring surgical intervention, intraoperative confirmation of spermatic cord torsion, and a definitive operative outcome of detorsion/orchiopexy or orchiectomy. To ensure homogeneity, patients with intrauterine testicular torsion and undescended testicular torsion were excluded. Patients with missing hematological parameters required for calculation of inflammatory indices were also excluded. The final analytical cohort comprised 165 patients Figure 1.

### 2.2. Operative Assessment and Outcome Definition

All patients underwent immediate scrotal exploration under general anesthesia. Following detorsion of the spermatic cord, the affected testis was wrapped in warm saline-soaked gauze and observed for at least 15 min to assess reperfusion. The intraoperative decision to preserve the testes or perform orchiectomy was made by the operating surgeon based on restoration of normal color, evidence of arterial pulsation, and parenchymal bleeding from the tunica albuginea after incision. Testes that remained dark or necrotic in appearance and lacked parenchymal bleeding despite reperfusion efforts were considered unsuitable for preservation and were managed with orchiectomy.

Long-term postoperative testicular atrophy was not used as the primary endpoint because follow-up data were not uniformly available for all preserved testes. Therefore, the primary outcome was restricted to the immediate intraoperative surgical decision at index exploration.

### 2.3. Data Collection and Variables

Clinical, timing, seasonal, laterality, and complete blood count variables were extracted from the hospital information system and operative records. Clinical variables included age, season, and side of torsion when available. Timing variables included symptom duration and emergency department waiting time. Symptom duration was defined as the interval from onset of scrotal pain to admission to the emergency department. Emergency department waiting time was defined according to the recorded in-hospital interval from emergency presentation to surgical evaluation or operative decision. When required for sensitivity analysis, total delay was calculated as symptom duration plus emergency department waiting time converted to hours.

Laboratory variables were derived from admission complete blood count parameters and included white blood cell count, neutrophil count, lymphocyte count, monocyte count, hemoglobin, platelet count, and mean platelet volume. Outcome labels were standardized before analysis to ensure consistent coding of orchiectomy and preservation/detorsion outcomes.

### 2.4. Derived Inflammatory Indices

All inflammatory indices were calculated directly from raw complete blood count variables within the analysis script before statistical modeling. The neutrophil-to-lymphocyte ratio was calculated as neutrophil count divided by lymphocyte count. The platelet-to-lymphocyte ratio was calculated as platelet count divided by lymphocyte count. The lymphocyte-to-monocyte ratio was calculated as lymphocyte count divided by monocyte count. The white blood cell-to-monocyte ratio was calculated as white blood cell count divided by monocyte count. The monocyte-to-mean platelet volume ratio was calculated as monocyte count divided by mean platelet volume. The hemoglobin-to-monocyte ratio was calculated as hemoglobin divided by monocyte count. The systemic inflammatory response index was calculated as neutrophil count multiplied by monocyte count divided by lymphocyte count. The aggregate index of systemic inflammation was calculated as neutrophil count multiplied by monocyte count and platelet count divided by lymphocyte count.

### 2.5. Statistical Analysis

Baseline characteristics were summarized according to operative outcome group. Continuous variables were reported as median and interquartile range. Categorical variables were reported as counts and percentages. Distributional characteristics of continuous variables were assessed graphically. Because most continuous variables were non-normally distributed, between-group comparisons were performed using the Mann–Whitney U test. Categorical variables were compared using Pearson’s chi-square test or Fisher’s exact test, as appropriate. All statistical tests were two-sided, and *p*-values below 0.05 were considered statistically significant. No formal sample size calculation was performed because of the retrospective design. The sample size was determined by the number of eligible surgically confirmed testicular torsion cases available during the study period after application of the prespecified exclusion criteria.

### 2.6. Feature Blocks for Predictive Modeling

Prespecified feature blocks were created to evaluate the relative contribution of timing, clinical, and inflammatory information. The timing block included symptom duration and emergency department waiting time. The clinical block included age, season, and side of torsion when available. The inflammatory block included raw complete blood count variables and inflammatory indices. The combined block included timing, clinical, raw complete blood count, and inflammatory variables.

Because total delay was derived from symptom duration and in-hospital waiting time, redundancy between timing variables was assessed before modeling. Symptom duration and total delay were not entered simultaneously in the same primary model when substantial collinearity was present. Models using total delay were treated as sensitivity analyses.

### 2.7. Machine-Learning Model Development and Validation

Five supervised classification algorithms were evaluated: logistic regression, random forest, XGBoost, LightGBM, and support vector machine. Logistic regression used L2 regularization and balanced class weighting. Random forest used balanced class weighting with conservative tuning of tree depth and minimum leaf size. XGBoost used a training-fold scale pos weight parameter to address class imbalance. LightGBM used balanced class weighting. Support vector machine used balanced class weighting with probability estimation enabled. Hyperparameter grids were compact and prespecified to reduce overfitting risk in this modest-sized retrospective cohort.

Model performance was estimated using nested stratified cross-validation. The outer loop consisted of stratified 5-fold cross-validation with shuffling and a random seed of 42. Within each outer training fold, hyperparameters were selected using stratified 3-fold cross-validation. All preprocessing steps were performed within the training data only. Numerical variables were imputed using the training-fold median. Categorical variables were imputed using the most frequent category and encoded using one-hot encoding. Standardization was applied for logistic regression and support vector machine models. No preprocessing, imputation, encoding, scaling, threshold selection, or hyperparameter tuning step used the outer validation fold.

Predicted probabilities from the outer validation folds were used to calculate model performance. Discrimination was assessed using receiver operating characteristic area under the curve and precision-recall area under the curve. Threshold-dependent metrics included accuracy, balanced accuracy, sensitivity for orchiectomy, specificity for preservation/detorsion, positive predictive value, negative predictive value, and F1-score. For threshold-dependent metrics, the probability threshold was selected within the training data only using Youden’s J statistic estimated from inner cross-validation predictions. Fold-level means and standard deviations were reported. Patient-level bootstrap 95% confidence intervals were calculated from out-of-fold predictions using 2000 bootstrap resamples.

Calibration was assessed using the Brier score, calibration intercept, calibration slope, and calibration curves based on out-of-fold predictions. Model interpretability was assessed using validation-based permutation importance in the combined feature block. Permutation importance was summarized as the mean decrease in receiver operating characteristic area under the curve after feature permutation across validation folds. SHAP analysis, when performed, was used only for post-validation interpretation and not for performance estimation.

All analyses were performed using Python 3.9.6 with pandas 2.3.3, NumPy 2.0.2, SciPy 1.13.1, scikit-learn 1.6.1, XGBoost 2.1.4, LightGBM 4.6.0, matplotlib 3.9.4, and SHAP 0.29.1. Results were reported as internally validated estimates from out-of-fold predictions. No independent external validation cohort was used.

## 3. Results

The final analytical cohort included 165 pediatric and adolescent patients who underwent surgical exploration for testicular torsion. Orchiectomy at index exploration was performed in 37 patients, whereas testicular preservation through detorsion and/or orchiopexy was performed in 128 patients.

Demographic and time-related variables according to operative outcome are presented in Table 1. Patients in the orchiectomy group were younger than those in the detorsion group (14.00 [12.00–15.50] vs. 15.00 [14.00–16.00] years, *p* = 0.027). Symptom duration was significantly longer in the orchiectomy group (48.00 [30.00–72.00] vs. 6.00 [2.00–24.00] h, *p* < 0.001). Emergency examination wait time, ultrasound wait time, and time to surgery did not differ significantly between groups. Total waiting time was also longer in the orchiectomy group (200.00 [160.00–304.00] vs. 190.00 [129.25–274.00] min, *p* < 0.001).

Complete blood count parameters are summarized in Table 2. Total white blood cell count, neutrophil count, lymphocyte count, eosinophil count, basophil count, platelet count, hemoglobin, and hematocrit did not differ significantly between the two groups. Monocyte count was higher in the orchiectomy group than in the detorsion group (0.92 [0.68–1.23] vs. 0.65 [0.50–0.93] × 10^9^/L, *p* < 0.001). Monocyte percentage was also higher in the orchiectomy group (7.93 [6.48–11.25] vs. 6.30 [4.30–7.65]%, *p* < 0.001). Among red cell and platelet indices, MCHC, RDW-CV, and MPV showed statistically significant differences between groups.

Hematological inflammatory indices are shown in Table 3. NLR did not differ significantly between groups (*p* = 0.381). MER was higher in the orchiectomy group (13.43 [6.99–30.56] vs. 7.57 [3.39–18.94], *p* = 0.014). PLR, derived from platelet and lymphocyte counts, did not differ significantly between groups (149.41 [99.89–192.66] vs. 127.92 [96.18–196.97], *p* = 0.604). LMR, WMR, and HMR were lower in the orchiectomy group, whereas MMPR, SIRI, and AISI were higher in the orchiectomy group; all of these differences were statistically significant.

**Figure 2:**
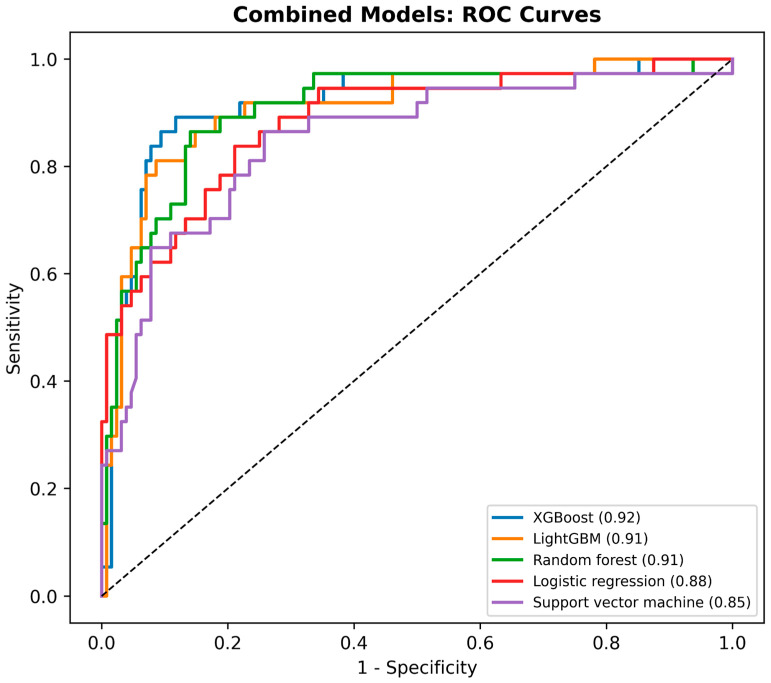
Receiver operating characteristic curves of the combined machine-learning models for predicting orchiectomy at index surgical exploration versus testicular preservation/detorsion at index exploration. ROC curves were generated from pooled out-of-fold predictions from internal cross-validation; tabulated performance is reported as fold-level mean ± SD.

The primary predictive analysis evaluated five supervised machine-learning algorithms within the Combined feature block, which incorporated timing variables, clinical characteristics, raw complete blood count parameters, and inflammatory indices. Among these models, XGBoost achieved the highest numerical discrimination, with a ROC-AUC of 0.926 ± 0.066 and a bootstrap 95% CI of 0.856–0.966 (Table 4). LightGBM and random forest demonstrated closely similar performance, with ROC-AUC values of 0.923 ± 0.044 and 0.921 ± 0.065, respectively. Logistic regression achieved a ROC-AUC of 0.884 ± 0.078, whereas support vector machine achieved a ROC-AUC of 0.859 ± 0.077. Out-of-fold ROC curves for the Combined models are shown in Figure 2.

Precision-recall analysis showed the highest PR-AUC for XGBoost at 0.841 ± 0.157, followed by random forest at 0.839 ± 0.145, logistic regression at 0.821 ± 0.109, LightGBM at 0.820 ± 0.139, and support vector machine at 0.761 ± 0.110 (Table 4). For threshold-dependent metrics, XGBoost achieved sensitivity of 0.839 ± 0.182, specificity of 0.851 ± 0.133, and F1-score of 0.727 ± 0.167. Random forest achieved sensitivity of 0.889 ± 0.186, specificity of 0.765 ± 0.223, and F1-score of 0.696 ± 0.230. Logistic regression achieved sensitivity of 0.782 ± 0.078, specificity of 0.812 ± 0.127, and F1-score of 0.663 ± 0.140. Precision-recall curves are provided in Supplementary Figure S1.

**Figure 3:**
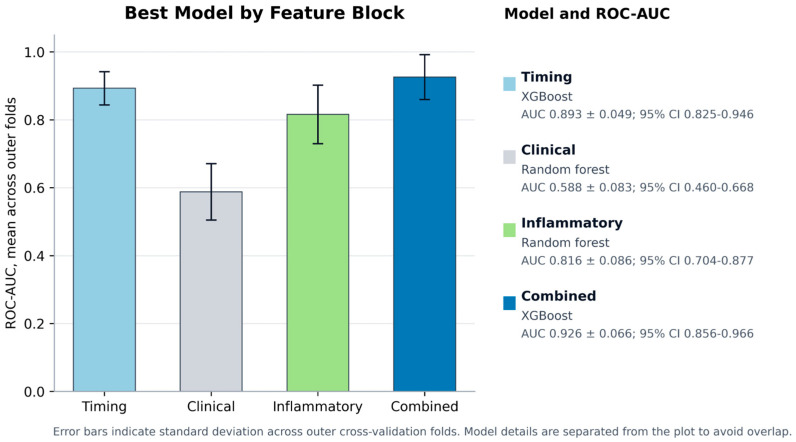
Feature-block comparison of the best-performing models for orchiectomy prediction.

Feature-block analysis was performed to evaluate the relative contribution of timing, clinical, inflammatory, and combined information. The best Timing model was XGBoost, with a ROC-AUC of 0.893 ± 0.049 and a 95% CI of 0.825–0.946 (Table 5). The Clinical block alone showed limited discrimination; the best Clinical model was random forest, with a ROC-AUC of 0.588 ± 0.083 and a 95% CI of 0.460–0.668. The best Inflammatory model was random forest, with a ROC-AUC of 0.816 ± 0.086 and a 95% CI of 0.704–0.877. The Combined block achieved the highest numerical discrimination, with XGBoost reaching a ROC-AUC of 0.926 ± 0.066 and a 95% CI of 0.856–0.966. Block-level model performance is shown in Figure 3.

**Figure 4:**
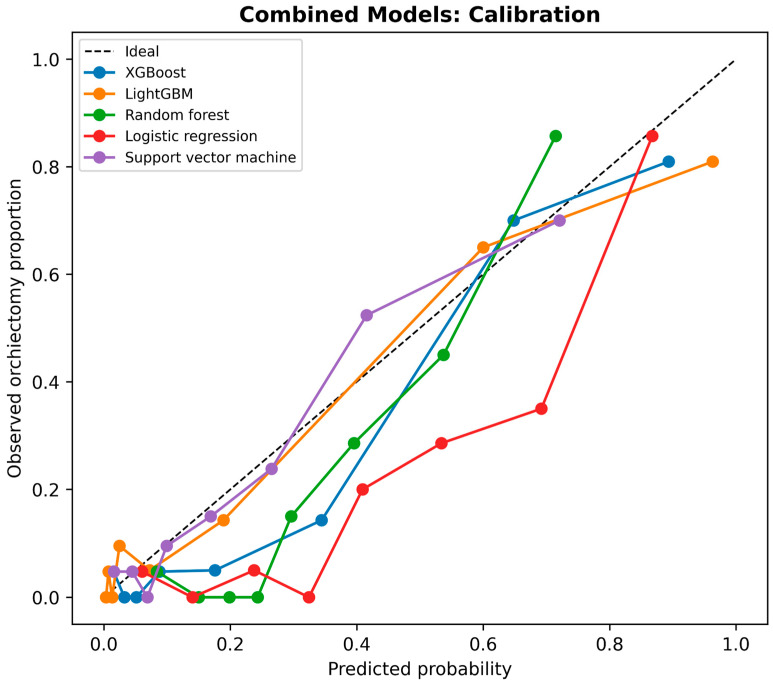
Calibration curves for the combined machine-learning models.

Calibration was assessed using out-of-fold predicted probabilities from the Combined models. XGBoost had the lowest Brier score, at 0.089 ± 0.051, followed by LightGBM at 0.091 ± 0.043, random forest at 0.114 ± 0.030, support vector machine at 0.117 ± 0.020, and logistic regression at 0.145 ± 0.041 (Table 4). Calibration intercepts and slopes are provided in Supplementary Table S3. Calibration curves for the Combined models are shown in Figure 4.

**Figure 5:**
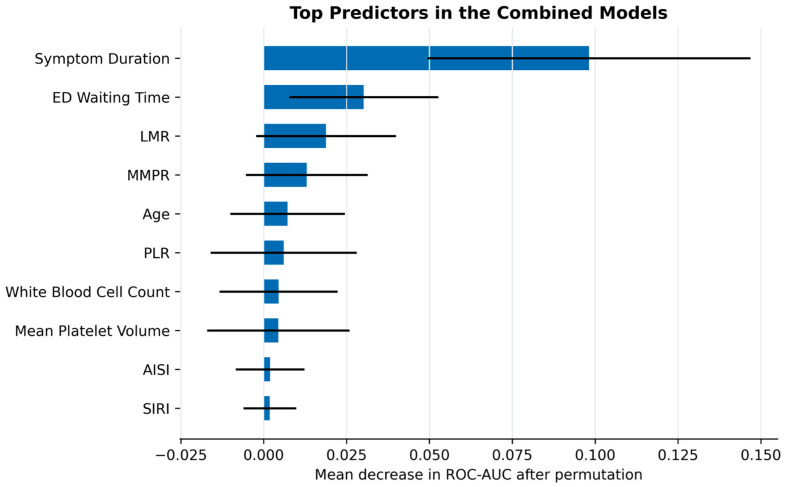
Validation-based permutation importance of the top predictors in the combined feature block.

Permutation-importance analysis was performed within the Combined feature block using validation-based decreases in ROC-AUC after feature permutation. Symptom duration was the highest-ranked predictor, followed by emergency department waiting time (Figure 5; Supplementary Table S1). Among laboratory and inflammatory variables, LMR, MMPR, PLR, white blood cell count, mean platelet volume, AISI, and SIRI were among the highest-ranked predictors.

SHAP analysis of the final Combined XGBoost model was performed as a post-validation interpretability analysis. Symptom duration had the highest mean absolute SHAP value, followed by emergency department waiting time and LMR. Additional contributing variables included mean platelet volume, WMR, MMPR, age, HMR, NLR, white blood cell count, PLR, and season. SHAP-based feature rankings are provided in Supplementary Table S2, with the corresponding importance plot and beeswarm plot shown in Supplementary Figures S2 and S3.

## 4. Discussion

This retrospective study on pediatric and adolescent testicular torsion (TT) demonstrated that the immediate surgical outcomes, specifically orchiectomy versus testicular preservation, are more accurately predicted through a combination of clinical and laboratory factors rather than relying on any single laboratory marker or clinical characteristic.

In alignment with prior research indicating that testicular salvage rates decrease as ischemic duration increases [2,4,5,6], our findings demonstrate that delayed presentation and extended symptom duration are the factors most significantly correlated with testicular non-viability and the subsequent requirement for orchiectomy (*p* < 0.001).

Consistent with existing evidence that salvage rates diminish as ischemic duration increases [2,4,5,6], the principal finding was that non-viability and subsequent orchiectomy were most strongly associated with delayed presentation and prolonged symptom duration (*p* < 0.001). Traditional teachings suggest that surgical detorsion within 6 h can achieve nearly 100% salvage, whereas beyond 12–24 h, the probability of a viable testis may decrease to approximately 20% [4,6]. While the literature reports emergency orchiectomy rates in TT ranging from 12% to 72.9%, our cohort required orchiectomy in 22.4% of cases, primarily driven by cumulative delays [6,8,14,15,16,17]. Feng et al. identified time to surgery and degree of torsion as predictors of testicular salvage in children, while Zvizdic et al. reported symptom duration as the only independent predictor of successful salvage in a pediatric case–control study [18,19]. Similar conclusions have been reported in pediatric hematological and nomogram-based prediction studies, in which symptom duration remained a central variable for salvage failure, necrosis, and orchiectomy [8,14,16,17]. Additionally, younger age, waiting time variables, and monocyte-centered inflammatory patterns provided crucial secondary risk information, confirming that a combined feature block offers superior predictive accuracy for testicular loss.

The waiting-time variables require a more nuanced interpretation. In conventional comparisons of our data, isolated emergency examination wait, ultrasound wait, and time to surgery were not uniformly significant, whereas total waiting time was longer in the orchiectomy group, and emergency department waiting time ranked highly in model interpretation. This pattern suggests that in-hospital timing may contribute to risk stratification when analyzed alongside symptom duration, age, and inflammatory variables, rather than functioning as a stand-alone predictor. Prior studies have linked delayed management, delayed diagnosis, misdiagnosis, and transfer-related pathways with adverse timing or outcome patterns in pediatric testicular torsion [20,21,22]. Therefore, the waiting time in the present model is best regarded as a workflow-related variable that may improve structured documentation, triage awareness, and care-pathway audit.

Younger age was also associated with orchiectomy in this cohort of patients. This signal was less dominant than symptom duration but remained clinically relevant. Yu et al. reported that delayed surgical management was more frequent among younger boys in a tertiary pediatric cohort, and Greear et al. described age-related differences in orchiectomy risk in a population including pediatric and adult patients. Shields et al. compared of torsion outcomes in boys younger than 13 years and those aged 13 years and older, found that the younger cohort exhibited a 46% orchiectomy rate, in contrast to 26% in post-pubescent adolescents [2,20,23]. Age may represent contextual/sociological vulnerability related to recognition, communication, or presentation delays rather than an isolated biological determinant of testicular viability.

The most consistent hematological signal in our cohort was monocyte-centered. The monocyte count and percentage were higher in the orchiectomy group. In addition, LMR, WMR, and HMR were lower, whereas MMPR, MER, SIRI, and AISI were higher in patients underwent orchiectomy. This pattern is consistent with prior pediatric studies in which monocyte count emerged as a clinically relevant hematological marker. Chen et al. reported that monocyte count was associated with testicular salvage outcome in a 12-year retrospective review, and their later pediatric nomogram again incorporated monocyte count together with symptom duration, intratesticular blood flow, and torsion degree [16,17]. Ekşi et al. also identified monocyte count among the variables contributing to orchiectomy prediction in a machine-learning model [13].

The monocyte-related indices derived in the present study should be interpreted as exploratory extensions of this signal. Direct evidence of pediatric torsion is strongest for monocyte count itself, while direct validation of LMR, WMR, HMR, MMPR, MER, and AISI in pediatric/adolescent torsion remains limited. Therefore, these markers should be considered as hypothesis-generating CBC-derived risk features rather than established biomarkers.

NLR and PLR were not significantly different between the orchiectomy and preservation groups in the univariable analysis. This finding does not exclude an inflammatory component in torsion outcomes but highlights the variable performance of conventional inflammatory ratios across cohorts, timing windows, and endpoint definitions. Jang et al. found that NLR predicted organ salvage, particularly in patients with marginal diagnostic delay, suggesting that its utility may depend on the clinical time window [7]. Delgado-Miguel et al. reported NLR as a predictor of orchiectomy or subsequent atrophy in a multicenter pediatric study, whereas Tian et al. incorporated PLR into a nomogram for pediatric salvage failure [8,24].

Several features of the current study may explain these differences in results. Our primary outcome was immediate intraoperative orchiectomy versus preservation, not a composite endpoint, including later testicular atrophy. Symptom duration showed a large separation between groups, potentially reducing the isolated univariable contribution of NLR or PLR. Furthermore, monocyte-centered variables may have captured a distinct aspect of the systemic response that is not reflected by neutrophil- or platelet-weighted ratios alone. Thus, NLR and PLR appear to be best understood as endpoint- and model-dependent variables rather than universally stable predictors.

The machine learning results support the incremental value of combining timing, clinical, and hematological information. The timing block performed strongly, the inflammatory block showed moderate discrimination, the clinical block alone was weak, and the combined block exhibited the highest performance. XGBoost produced the best numerical ROC-AUC, with LightGBM and random forest as close competitors. This pattern is compatible with the possibility that nonlinear relationships and interactions among delay, age, and inflammatory features contributed to the prediction. In this sense, the present findings reinforce a broader clinical concept: risk prediction in acute surgical disease is most informative when it mirrors real medical reasoning synthesizing time, clinical context, and biological response rather than reducing decision-making to a single variable.

Direct machine learning evidence for predicting testicular torsion outcomes remains limited. Ekşi et al. compared a machine learning model with a conventional statistical approach for orchiectomy prediction and reported favorable performance for random forest [13]. Concio et al. provided recent proof-of-concept evidence for ML-based post-torsion viability prediction, although their endpoint differed from immediate intraoperative orchiectomy versus preservation [25]. In parallel, prediction studies by Chen, Tian, and Mao demonstrate that models integrating clinical, imaging, and hematological variables can predict salvage failure or orchiectomy in pediatric torsion [8,14,16]. Compared with this literature, the present analysis adds multi-algorithm comparison, feature-block evaluation, PR-AUC reporting, calibration assessment, and SHAP/permutation-based interpretability. However, the model was only internally validated, and no independent external validation cohort was available.

Interpretability analyses supported the clinical plausibility of the model. Symptom duration was the highest-ranked feature in both permutation importance and SHAP analyses, mirroring the most consistent predictor in the literature [8,14,16,17,18,19]. The emergency department waiting time and selected inflammatory variables were followed. Importantly, these findings indicate that the model’s performance was driven by clinically interpretable variables, supporting its biological plausibility rather than relying on opaque or incidental predictors of outcomes.

The clinical role of this model is risk stratification rather than decision replacement. Suspected testicular torsion remains a surgical emergency, and no ML model should delay exploration or override the intraoperative assessment of reperfusion, color, arterial pulsation, or parenchymal bleeding. If externally validated, a model of this type could support triage documentation, family counseling, prioritization of high-risk presentations, audit of care pathways, and follow-up planning after attempted salvage.

This study had several limitations. This was a retrospective, single-center study, with a modest number of orchiectomy events. The endpoint was immediate operative management, not long-term testicular atrophy, endocrine function or fertility. This distinction is important because late testicular loss may occur after the initial salvage [6]. The torsion degree and detailed Doppler variables were not fully integrated into the primary model, although prior prediction studies have demonstrated their prognostic value [8,14,16]. Finally, despite nested cross-validation, bootstrap confidence intervals, calibration analysis, and interpretability assessment, the absence of external validation limits generalizability. Future studies should validate these findings in multicenter pediatric cohorts, include standardized torsion degree and Doppler variables, evaluate long-term atrophy after preservation, and compare interpretable regression-based models with modern ML algorithms. Monocyte-centered indices, particularly LMR, WMR, HMR, MMPR, MER, and AISI, require independent validation before they can be used in clinical settings.

## 5. Conclusions

In conclusion, immediate orchiectomy was most strongly associated with prolonged symptom duration, whereas younger age, waiting time variables, and monocyte-centered inflammatory patterns provided complementary risk information. NLR and PLR were not significant as isolated univariable markers, emphasizing the endpoint-dependent nature of the inflammatory ratios. The combined XGBoost model showed strong internal discrimination and clinically coherent interpretability; however, its current role is limited to risk stratification and triage support. External validation is required before clinical application.

## Figures and Tables

**Table 1:** Comparison of demographic and time-related variables between the orchiectomy and detorsion groups.

Variable	Orchiectomy (*n* = 37) Median [IQR]	Detorsion (*n* = 128) Median [IQR]	*p*-Value
Age (years)	14.00 [12.00–15.50]	15.00 [14.00–16.00]	0.027
Symptom duration (h)	48.00 [30.00–72.00]	6.00 [2.00–24.00]	<0.001
Emergency examination wait time (min)	7.00 [4.25–18.00]	5.00 [2.00–12.00]	0.097
US wait time (min)	26.50 [19.25–46.75]	29.50 [20.00–55.50]	0.409
Time to surgery (min)	162.00 [109.00–185.00]	141 [95.00–206.00]	0.805
Total waiting time (min)	200.00 [160.00–304.00]	190.00 [129.25–274.00]	<0.001

**Table 2:** Comparison of complete blood count parameters between the orchiectomy and detorsion groups.

Variable	Hematology Unit	Orchiectomy (*n* = 37) Median [IQR]	Detorsion (*n* = 128) Median [IQR]	*p*-Value
White blood cell count (WBC)	(×10^9^/L)	11.90 [8.90–15.4]	11.75 [9.4–13.60]	0.924
Neutrophils count	(×10^9^/L)	9.21 [6.00–10.88]	8.63 [5.91–10.95]	0.688
Neutrophils	(%)	70.40 [61.30–77.50]	75.55 [62.12–82.65]	0.152
Lymphocytes count	(×10^9^/L)	2.06 [1.43–2.60]	1.92 [1.41–2.74]	0.991
Lymphocytes	(%)	17.92 [12.55–23.57]	17.00 [11.82–27.77]	0.743
Monocytes count	(×10^9^/L)	0.92 [0.68–1.23]	0.65 [0.50–0.93]	<0.001
Monocytes	(%)	7.93 [6.48–11.25]	6.30 [4.30–7.65]	<0.001
Eosinophils count	(×10^9^/L)	0.05 [0.03–0.10]	0.08 [0.02–0.16]	0.322
Eosinophils	(%)	0.60 [0.20–1.00]	0.60 [0.30–1.57]	0.281
Basophils count	(×10^9^/L)	0.05 [0.02–0.06]	0.04 [0.03–0.07]	0.496
Basophils (%)	(%)	0.40 [0.30–0.58]	0.40 [0.30–0.60]	0.885
NRBC	(×10^9^/L)	0.00 [0.00–0.00]	0.00 [0.00–0.00]	0.779
NRBC (%)	(%)	0.00 [0.00–0.04]	0.00 [0.00–0.00]	0.215
Red blood cell count (RBC)	(×10^9^/L)	5.04 [4.89–5.42]	5.10 [4.85–5.33]	0.839
Hemoglobin	g/dL	14.26 [12.92–15.30]	14.50 [13.25–15.00]	0.618
Hematocrit	(%)	42.00 [37.71–44.20]	41.15 [38.55–43.09]	0.545
Mean corpuscular volume (MCV)	fL	80.85 [77.20–84.40]	80.35 [76.77–84.32]	0.715
Mean corpuscular hemoglobin (MCH)	pg	27.77 [26.52–29.00]	28.20 [26.70–29.77]	0.492
Mean corpuscular hemoglobin concentration (MCHC)	g/dL	34.05 [33.10–34.87]	34.71 [33.90–35.90]	0.009
RDW-SD	fL	37.85 [24.77–39.28]	37.20 [35.45–38.92]	0.498
RDW-CV	%	13.20 [12.87–13.95]	12.80 [12.25–13.55]	0.049
Platelet count	(×10^9^/L)	289.00 [231.50–347.00]	276.50 [227.25–317.75]	0.409
Mean platelet volume (MPV)	fL	9.15 [7.85–9.80]	9.60 [8.62–10.10]	0.031
Plateletcrit (PCT)	%	0.27 [0.25–0.34]	0.27 [0.23–0.30]	0.368
Platelet distribution width (PDW)	fL	11.35 [10.40–15.12]	11.00 [9.70–12.25]	0.340

**Table 3:** Comparison of recalculated hematological inflammatory indices between the orchiectomy and detorsion groups.

Variable	Formula	Orchiectomy (*n* = 37) Median [IQR]	Detorsion (*n* = 128) Median [IQR]	*p*-Value
Neutrophil-to-lymphocyte ratio (NLR)	Neutrophils count/Lymphocytes count	3.97 [2.54–5.85]	4.42 [2.28–6.82]	0.381
Monocyte-to-eosinophil ratio (MER)	Monocytes count/Eosinophils count	13.43 [6.99–30.56]	7.57 [3.39–18.94]	0.014
Platelet-to-lymphocyte ratio (PLR)	Platelet count/Lymphocytes count	149.41 [99.89–192.66]	127.92 [96.18–196.97]	0.604
Lymphocyte-to-monocyte ratio (LMR)	Lymphocytes count/Monocytes count	2.01 [1.76–2.88]	3.20 [2.18–4.24]	<0.001
White blood cell-to-monocyte ratio (WMR)	White blood cell count/Monocytes count	12.59 [8.69–14.51]	15.74 [13.26–23.07]	<0.001
The monocyte/mean platelet volume (MMPR)	Monocytes count/Mean platelet volume	0.11 [0.08–0.15]	0.07 [0.05–0.10]	<0.001
Hemoglobin-to-monocyte ratio (HMR)	Hemoglobin/Monocytes count	13.20 [10.67–19.30]	21.67 [15.29–28.60]	<0.001
Systemic inflammation response index (SIRI)	Neutrophils count X Monocytes count/Lymphocytes count	3.60 [2.33–5.86]	2.62 [1.51–4.52]	0.022
Aggregate index of systemic inflammation (AISI)	Neutrophils count X Monocytes count X Platelet count/Lymphocytes count	1052.60 [702.43–1908.48]	745.12 [407.54–1314.71]	0.021

**Table 4:** Internally validated performance of supervised machine-learning models in the combined feature block.

Model	ROC-AUC Mean ± SD	ROC-AUC 95% CI	PR-AUC Mean ± SD	PR-AUC 95% CI	Sensitivity Mean ± SD	Sensitivity 95% CI	Specificity Mean ± SD	Specificity 95% CI	Accuracy Mean ± SD	Accuracy 95% CI	F1 Mean ± SD	F1 95% CI	Brier Mean ± SD	Brier 95% CI
LightGBM	0.923 ± 0.044	0.846–0.959	0.820 ± 0.139	0.587–0.894	0.693 ± 0.195	0.553–0.842	0.842 ± 0.186	0.778–0.903	0.812 ± 0.118	0.752–0.873	0.636 ± 0.107	0.500–0.740	0.091 ± 0.043	0.058–0.127
Logistic regression	0.884 ± 0.078	0.807–0.941	0.821 ± 0.109	0.643–0.875	0.782 ± 0.078	0.636–0.913	0.812 ± 0.127	0.741–0.874	0.806 ± 0.102	0.745–0.861	0.663 ± 0.140	0.521–0.747	0.145 ± 0.041	0.121–0.172
Random forest	0.921 ± 0.065	0.841–0.958	0.839 ± 0.145	0.630–0.885	0.889 ± 0.186	0.781–0.976	0.765 ± 0.223	0.689–0.837	0.794 ± 0.176	0.727–0.855	0.696 ± 0.230	0.543–0.761	0.114 ± 0.030	0.095–0.135
Support vector machine	0.859 ± 0.077	0.762–0.920	0.761 ± 0.110	0.548–0.821	0.675 ± 0.074	0.515–0.824	0.813 ± 0.116	0.742–0.876	0.782 ± 0.089	0.715–0.842	0.591 ± 0.104	0.444–0.697	0.117 ± 0.020	0.086–0.149
XGBoost	0.926 ± 0.066	0.856–0.966	0.841 ± 0.157	0.596–0.892	0.839 ± 0.182	0.707–0.947	0.851 ± 0.133	0.791–0.911	0.848 ± 0.103	0.794–0.903	0.727 ± 0.167	0.600–0.812	0.089 ± 0.051	0.062–0.120

Metrics are reported from out-of-fold predictions during internal cross-validation. Values are fold-level mean ± SD with patient-level bootstrap 95% confidence intervals where applicable.

**Table 5:** Feature-block analysis comparing timing, clinical, inflammatory, and combined predictors for orchiectomy prediction.

Block	Model	ROC-AUC Mean ± SD	ROC-AUC 95% CI
Timing	XGBoost	0.893 ± 0.049	0.825–0.946
Clinical	Random forest	0.588 ± 0.083	0.460–0.668
Inflammatory	Random forest	0.816 ± 0.086	0.704–0.877
Combined	XGBoost	0.926 ± 0.066	0.856–0.966
Combined	Logistic regression	0.884 ± 0.078	0.807–0.941

## Data Availability

The datasets generated and analyzed during the current study are available from the corresponding author upon reasonable request.

## Supplementary Materials

The following supporting information can be downloaded at: https://www.mdpi.com/article/10.3390/diagnostics16132034/s1, Table S1. Validation-based permutation importance of predictors in the combined feature block. Table S2. SHAP-based feature ranking for the combined XGBoost model. Table S3. Calibration metrics for the combined machine-learning models. Figure S1. Precision-recall curves for the combined machine-learning models. Figure S2. SHAP importance plot for the combined XGBoost model. Figure S3. SHAP summary beeswarm plot for the combined XGBoost model.
