# Supplementary material for: Is It Too Late? Machine Learning for Predicting Orchiectomy Versus Testicular Preservation in Acute Torsion
Source: Diagnostics (Basel). 2026 Jun 29;16(13):2034. doi: 10.3390/diagnostics16132034 (PMC13359506; doi:10.3390/diagnostics16132034)

Supplementary Material

Supplementary Table S1. Validation-based permutation importance of predictors in the combined feature block.

Predictor	Mean importance	SD	Median rank	Top-10 frequency
Symptom Duration	0.098	0.049	1	0.96
ED Waiting Time	0.030	0.022	2	0.92
LMR	0.019	0.021	4	0.80
MMPR	0.013	0.018	6	0.72
Age	0.0072	0.017	8	0.68
PLR	0.0060	0.022	11	0.48
White Blood Cell Count	0.0045	0.018	9	0.52
Mean Platelet Volume	0.0044	0.021	8	0.60
AISI	0.0019	0.010	12	0.40
SIRI	0.0018	0.0080	11	0.48
NLR	0.0011	0.013	12	0.48
WMR	0.0011	0.019	8	0.56
Lymphocyte Count	0.0007	0.0085	11	0.48
Side	0.0003	0.0064	12	0.44
Season	0.0002	0.0081	10	0.52
Monocyte Count	0.0002	0.0090	12	0.44
Platelet Count	-0.0001	0.012	10	0.52
Neutrophil Count	-0.0002	0.0066	13	0.32
HMR	-0.0017	0.011	11	0.32
Hemoglobin	-0.0026	0.0088	11	0.48

Note. Permutation importance was summarized as the mean decrease in ROC-AUC after feature permutation across validation folds. Top-10 frequency indicates the proportion of estimates in which the predictor was ranked among the top 10.

Supplementary Table S2. SHAP-based feature ranking for the combined XGBoost model.

Predictor	Mean absolute SHAP value
Symptom Duration	1.132
ED Waiting Time	0.614
LMR	0.415
Mean Platelet Volume	0.267
WMR	0.248
MMPR	0.215
Age	0.197
HMR	0.095
NLR	0.092
White Blood Cell Count	0.061
PLR	0.046
Season: Spring	0.041
Platelet Count	0.039
Monocyte Count	0.029
Hemoglobin	0.026
AISI	0.022
SIRI	0.017
Season: Winter	0.017
Neutrophil Count	0
Season: Autumn	0
Lymphocyte Count	0
Season: Summer	0
Side: Left	0
Side: Right	0

Supplementary Table S3. Calibration metrics for the combined machine-learning models.

Block	Model	Brier score	Calibration intercept	Calibration slope
Combined	LightGBM	0.091	-0.399	0.659
Combined	Logistic regression	0.145	-1.338	1.457
Combined	Random forest	0.114	-0.305	2.141
Combined	Support vector machine	0.117	0.0073	0.998
Combined	XGBoost	0.089	-0.563	1.050

Note. Calibration metrics were calculated using out-of-fold predicted probabilities from the combined feature-block models.

Supplementary Figure S1. Precision-recall curves for the combined machine-learning models.

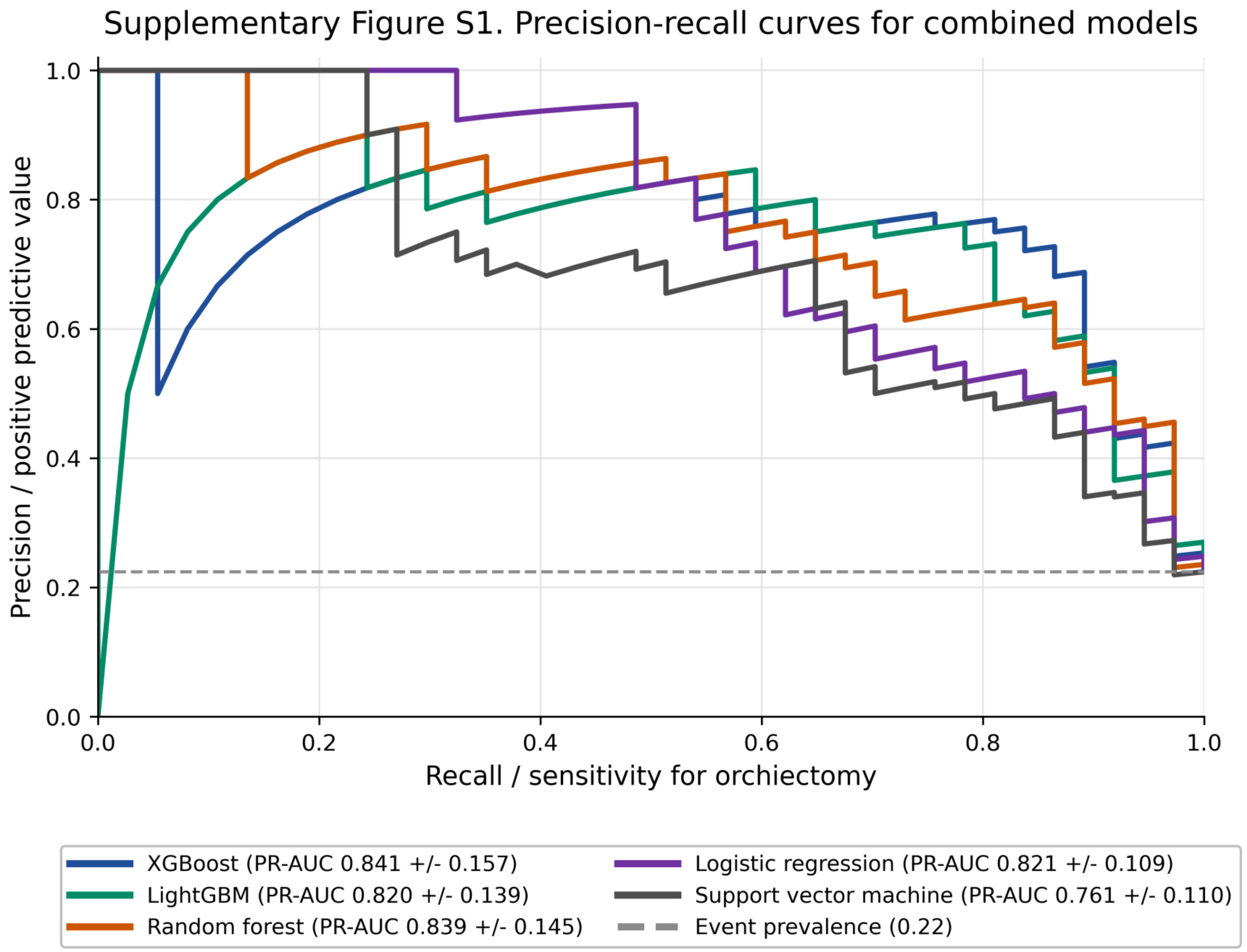

Supplementary Figure S2. SHAP importance plot for the combined XGBoost model.

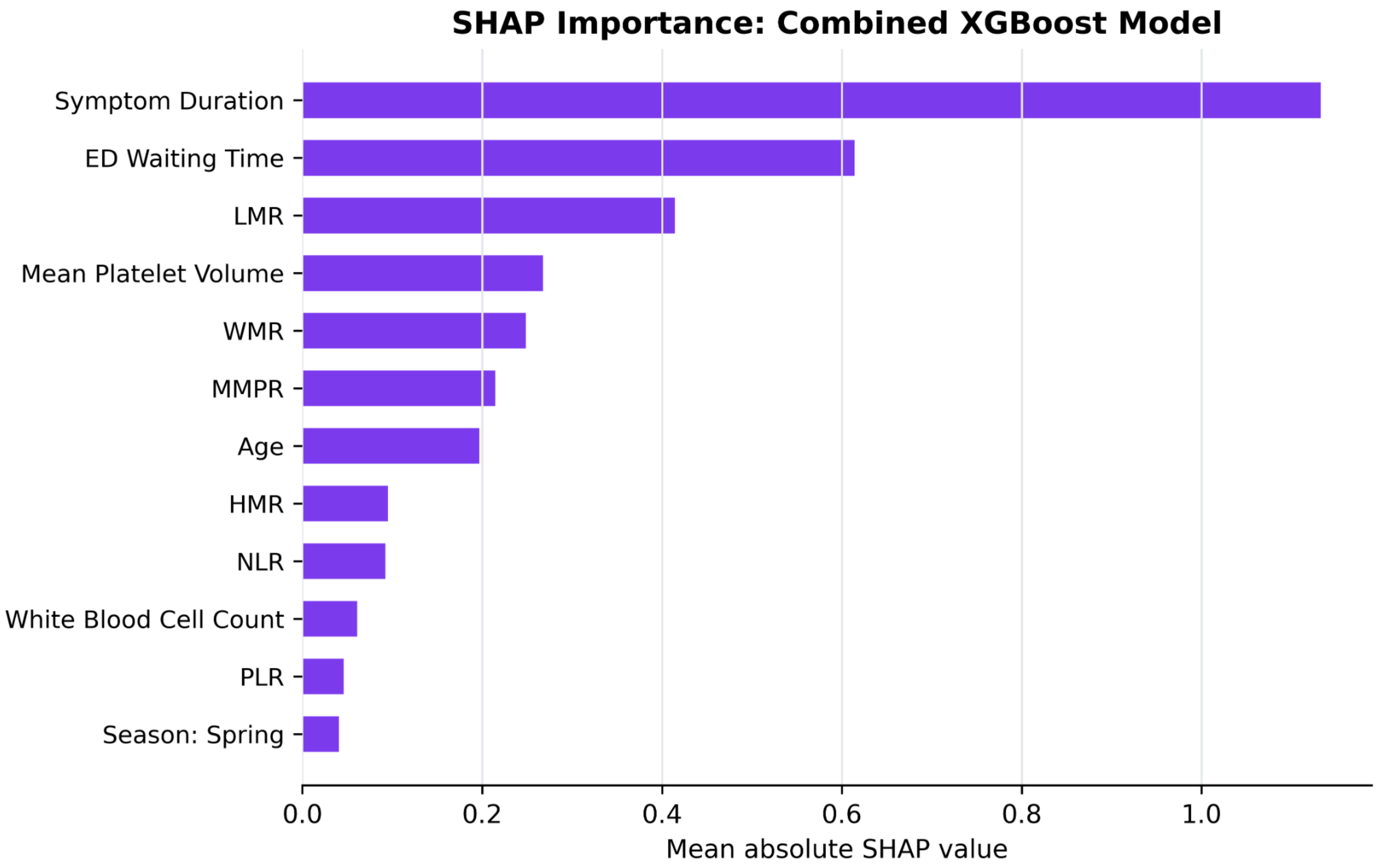

Supplementary Figure S3. SHAP summary beeswarm plot for the combined XGBoost model.

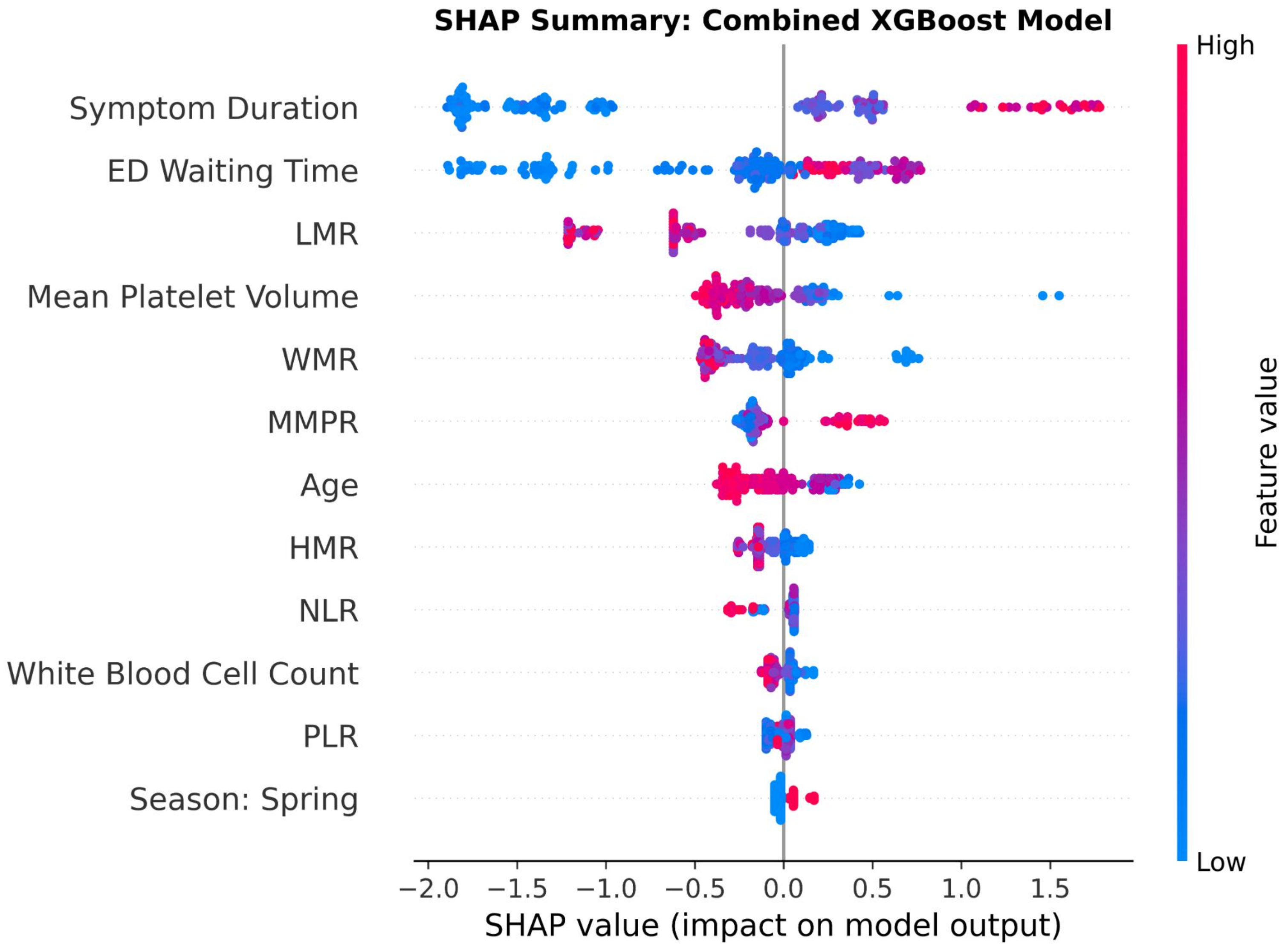

Supplement: Supplementary file 1 [file diagnostics-16-02034-s001.zip › diagnostics-4293992-supplementary.pdf]
